# Can’t touch this: *the emergence of contactless sleep technology*

**DOI:** 10.1093/sleep/zsae207

**Published:** 2024-10-04

**Authors:** Alexander Yoo, Ron C Anafi

**Affiliations:** Department of Medicine, Chronobiology and Sleep Institute, Perelman School of Medicine, University of Pennsylvania, Philadelphia, PAUSA; Department of Medicine, Chronobiology and Sleep Institute, Perelman School of Medicine, University of Pennsylvania, Philadelphia, PAUSA

For centuries, astronomers peered at the night sky through ground-based telescopes, capturing fleeting glimpses of celestial phenomena during clear nights. The launch of orbiting telescopes and the advent of wide-field sky surveys marked a paradigm shift [1], enabling continuous observation of the cosmos. The transition from episodic to persistent monitoring unveiled dynamic processes and rare events previously hidden from view. Closer to home, new technologies including ambulatory blood pressure monitors and long-term event monitors are allowing cardiologists to identify clinically important patterns in heart function. In sleep science, a similar transformation appears on the horizon.

The gold standard for sleep assessment, polysomnography (PSG), provides detailed information about sleep stages and physiology [2], but it comes with significant limitations. PSG requires that patients sleep in a laboratory setting, making it impractical for extended studies. Artificial laboratory conditions likely alter sleep architecture and skew these data, particularly in children [3]. Our understanding of human sleep and its changing character over time has developed from “stitching together” snapshots taken under artificial conditions. Extended, longitudinal studies that characterize sleep with high temporal resolution in non-laboratory settings have the potential to reshape our understanding of sleep and its relationship with both healthy aging and the progression of disease.

However, assessing sleep physiology and architecture over extended periods remains a challenge. Actigraphy and similar technologies allow for prolonged observations. But without adding additional sensors they lack the ability to accurately distinguish between sleep stages [4], characterize electrophysiological sleep features, or detect and classify respiratory events. These constraints have left gaps in our understanding. We know little about how sleep changes in response to life events, environmental changes, disease progression, or treatment. The work by He and colleagues reported in this issue of *SLEEP*, “What Radiowaves Tell Us About Sleep” [5], represents a significant advance in this quest.

He and colleagues present a machine-learning model that stages sleep and scores respiratory events using breathing patterns. Chest movements and thus breathing patterns are detected through low-power, high-frequency radio waves reflected off a sleeping person’s body. A deep neural network processes these nocturnal breathing signals to generate sleep hypnograms and detect apnea–hypopnea events. Trained on data from thousands of sleep studies in public and private repositories, the authors report results that reasonably approximate the gold standard. By employing techniques like knowledge distillation from electroencephalography (EEG) data and classifier retraining, the model returns high-accuracy predictions and equitable performance across diverse demographics and health conditions. This contactless technology promises to monitor for sleep apnea and assess sleep architecture with minimal impact on the patient.

The authors’ decision to validate their algorithm, submit their work for peer review, and share their code with the scientific community is commendable. These essential, confidence-building steps are often overlooked in a fast-paced marketplace where consumers trust “scientific sounding” algorithms. Many devices fail to gain traction among researchers and clinicians due to a lack of transparency regarding their inner workings and by extension, their limitations. By contrast, the work of He et al. facilitates replication and encourages further innovation. Reassuringly, they are not alone. Other academic groups have similarly subjected their deep learning models to peer scrutiny [6, 7]. While this trend is less common in the industry, a consumer smartwatch recently obtained FDA approval for its sleep apnea assessment [8, 9]. Transparency will be critical in cultivating trust in new, increasingly complex, sleep monitoring approaches.

The applications of automated, contactless technologies are wide-ranging. There is, for example, increasing interest in pharmacological treatments for obstructive sleep apnea (OSA) [10]. Contactless monitoring could facilitate robust clinical trials. Nearable technologies reduce the impact on study participants, allowing them to sleep in their own homes without cumbersome equipment. For investigators, technologies with automated analysis can streamline the collection of sleep data from large, extended studies. If pharmacological treatments for OSA become available, this same technology could enable long-term efficacy monitoring much like the reports generated by current positive airway pressure devices.

In the current study, the machine learning model was trained to replicate traditional sleep staging and the currently accepted apnea–hypopnea index (AHI). But our sleep staging system owes much to historical convention. Similarly, while AHI remains the primary metric used to assess sleep apnea, it has been shown to be a poor predictor of sleepiness [11]. Furthermore, several randomized CPAP trials have not shown a survival benefit when patients are selected using standard AHI criteria [12]. Thus, one of the greatest potential benefits of contactless technologies may be their enabling advancement beyond our current metrics. By collecting long-term sleep data in a noninvasive fashion, future researchers may be able to identify novel features from raw signals that more directly predict specific health outcomes or treatment responses. For example, in diabetes management, continuous glucose monitoring has birthed the “time in range” metric, which may potentially supplant the traditional HbA1c measure [13].

While these future applications are exciting, the technology has more immediate relevance. Polysomnography can be difficult to tolerate, especially for children and those with neurodevelopmental conditions [14]. Contactless technologies offer a comfortable alternative, reducing the impacts imposed on families. Contactless monitoring which accurately determines sleep onset could similarly inform insomnia therapies assessing both treatment adherence and efficacy. Cognitive behavioral recommendations for insomnia could be tailored to a patient’s treatment responses. The evaluation of hypersomnia would be aided by confirming that patients are getting sufficient nightly sleep.

The utility of continuous sleep monitoring likely extends beyond sleep disorders. For instance, prodromal sleep disturbances commonly herald mood disturbances in patients with bipolar or unipolar depression [15]. Sleep monitoring could act as an early warning system. The intricate relationship between sleep and neurodegenerative diseases [16] highlights another potential application. Indeed, the current publication builds on work from the same team diagnosing Parkinson’s disease using radio wave-based breathing assessments in sleeping patients [17]. Changes in sleep architecture may also be associated with disease progression [18]. Continuous, contactless monitoring in high-risk individuals could prompt further evaluation.

Despite the clear potential, technical hurdles and broader scoping concerns will need to be addressed as these technologies are adopted. While initial results show promise across diverse demographic groups, validation is needed for populations where accuracy may be compromised. For example, our own clinical experience suggests that respiratory effort measurements are more fraught in morbidly obese individuals. Similar concerns surround those with some neuromuscular conditions. Moreover, He et al. analyzed data collected in a controlled laboratory setting. Environmental factors in homes or imperfect home installations may interfere with outpatient measurements. While the authors of the study suggest that the presence of a bed partner will not influence the radio wave measurements, this too should be thoroughly tested.

Beyond these technical concerns, continuous monitoring in a private environment brings ethical and privacy issues to the fore, raising questions about data security and consent [19]. Moreover, if this technology enters the consumer market, It may contribute to the over-medicalization of sleep, where normal variations are misinterpreted as problematic. Additionally, there is the potential to misapply scientific findings when interpreting personal sleep data [20]. These misapplications could result in unwarranted conclusions, stirring unnecessary anxiety. The challenge ahead lies in leveraging the clear advantages of contactless continuous monitoring while being mindful of the potential hazards—a balance that researchers, clinicians, and consumers alike will need to navigate as this technology matures.
